# Echoes from Within: Mapping Gastrointestinal Obstruction with Ultrasound

**DOI:** 10.3390/diagnostics15192511

**Published:** 2025-10-02

**Authors:** Lior Abramson, Rebecca G. Theophanous, Brice Lefler, Lindsey Wu, Amber L. Bowman, Jacqueline K. Olive, Yuriy S. Bronshteyn

**Affiliations:** 1 Durham VA Medical Center, Durham, NC 27705, USA; lior.abramson@duke.edu (L.A.); rebecca.theophanous@duke.edu (R.G.T.); brice.lefler@duke.edu (B.L.); lindsey.wu@duke.edu (L.W.); amber.bowman@duke.edu (A.L.B.); 2 Department of Medicine, Duke University School of Medicine, Durham, NC 27710, USA; 3 Department of Emergency Medicine, Duke University School of Medicine, Durham, NC 27710, USA; 4 Division of Cardiovascular and Thoracic Surgery, Department of Surgery, Duke University School of Medicine, Durham, NC 27710, USA; jacqueline.olive@duke.edu; 5 Division of Trauma, Acute, and Critical Care Surgery, Department of Surgery, Duke University School of Medicine, Durham, NC 27710, USA; 6 Department of Anesthesiology, Duke University School of Medicine, Durham, NC 27710, USA

**Keywords:** point-of-care-ultrasound, bowel ultrasound, gastric ultrasound, small bowel obstruction, pneumoperitoneum, ileus

## Abstract

Patients presenting with abdominal pain and/or distension require rapid diagnostics to narrow the differential diagnosis from a long list of obstructive gastrointestinal (GI) pathologies that may appear clinically similar but warrant distinct management. While the workup of abdominal distension currently centers around computed tomography (CT), this modality is costly, requires radiation exposure, and necessitates patient transport, potentially delaying care. In contrast, point-of-care ultrasound (POCUS) avoids ionizing radiation and the need for patient transport while providing some insight into the gastrointestinal size and function. While POCUS cannot currently replace CT in the definitive diagnosis of GI obstructive pathologies, it remains a promising tool to help with the initial triage and monitoring responses to therapy for several causes of functional and/or mechanical GI obstruction, such as gastric dilation, ileus, and small bowel obstruction. Because the obstruction severity and features can evolve over time, POCUS enables serial examinations to monitor the progression or resolution. This manuscript reviews characteristic sonographic findings that help distinguish obstructive GI conditions and highlights practical techniques for integrating gastric and intestinal POCUS to improve diagnostic accuracy and expedite treatment.

**Figure 1:**
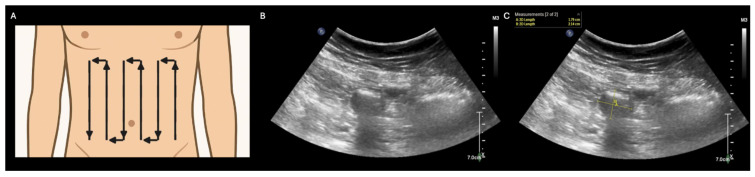
Abdominal pain and/or distension are common presenting complaints in a variety of clinical settings [1,2,3,4]. Traditionally, identifying the etiology of these signs/symptoms has required imaging modalities that utilize ionizing radiation (e.g., X-ray or computed tomography). However, these modalities have significant limitations, including but not limited to the need for patient transport (sometimes including inter-facility transfer) [5] and an oncologic risk to patients [6,7]. These limitations could be avoided by an imaging modality that can both be performed at the point of care and avoids ionizing radiation. Fortunately, point-of-care ultrasound (POCUS) of the GI tract is emerging as just such a bedside tool. While GI POCUS currently lacks the diagnostic accuracy of a CT scan, it may nevertheless help with the initial triaging of GI dysfunction and monitoring the response to therapy over time [8]. This manuscript presents a series of illustrative POCUS images and clips that are representative of important causes of GI dysfunction: ileus, small bowel obstruction, gastric dilation, perforated viscus, and colonic dilation/dysfunction. POCUS screening for GI dysfunction can be performed using a low-frequency (e.g., curvilinear) transducer with a two-phase approach as described by Abramson, et al.: (1) the “lawnmower technique” to scan the anterior abdominal wall systematically, screening primarily for small bowel dilatation (**A**) and (2) gastric evaluation in the subxiphoid and left upper quadrant regions to screen for gross gastric dilation (Figure 2) [5]. In the first phase of the protocol (**A**), a low-frequency (typically curvilinear) transducer is used to perform a systematic scan (“lawnmower technique”) across the ventral abdomen, beginning with an initial probe placement in any abdominal quadrant and then the translation of the probe across the abdomen in vertical lines to evaluate the entire anterior abdominal wall and bilateral abdominal flanks. During this systematic scan, the small bowel is examined primarily to assess its (i) diameter and (ii) peristalsis. A normal small bowel has a maximal outer-wall to outer-wall diameter of ≤ 2.5 cm, with values between 2.5 and 2.75 considered borderline abnormal [8] (**B**,**C**), and displays waves of peristalsis where the bowel lumen periodically collapses and re-expands (see Supplementary Video S1 for dynamic visualization of small bowel peristalsis).

**Figure 2:**
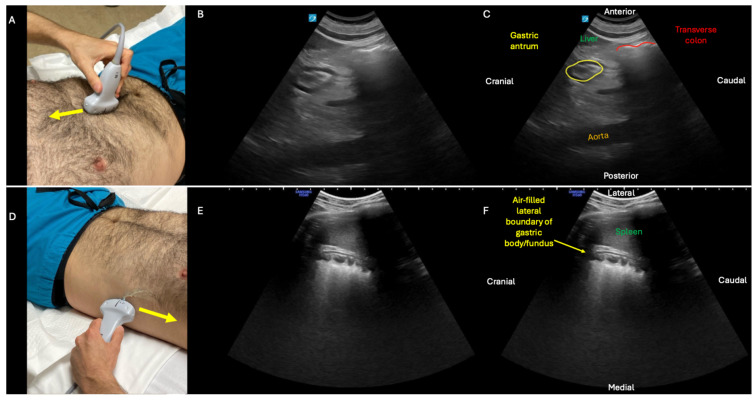
In the second phase of the GI POCUS protocol, two gastric ultrasound views are obtained: subxiphoid and left upper quadrant (LUQ). **Top panel**: subxiphoid view obtained with low-frequency, curvilinear probe positioned in approximately the mid-sagittal plane just caudal to xiphoid process (**A**), with probe indicator pointing cranially (yellow arrow), and example of an antrum (**B**,**C**) with trace fluid and no gross solids. Although the subxiphoid view is initially performed with patient supine, the sensitivity of the exam for gastric distension is increased by turning the patient right lateral decubitus and repeating this view [9]. **Bottom panel**: LUQ view of gastric body/fundus obtained with probe placed along the left flank at approximately the mid-axillary line (**D**), probe indicator pointing cranially (yellow arrow), view centered on spleen, and probe angled anteriorly to visualize stomach body/fundus rather than kidney (which can be visualized with this same probe positioning but with probe angled posteriorly). In this LUQ view, when the stomach is empty, the typical findings are acoustic shadowing within the body/fundus (**E**,**F**) due to the normal presence of air in the stomach (see also Supplementary Video S2; contrast with Figure 7 and Supplementary Video S7 showing gastric distension). By combining intestinal (Figure 1) and gastric (Figure 2) ultrasound, this two-phase protocol generates data that can help sort patients into categories of normal versus abnormal function of the stomach, small bowel, and—to a lesser degree—large bowel (see Figure 8).

**Figure 3:**
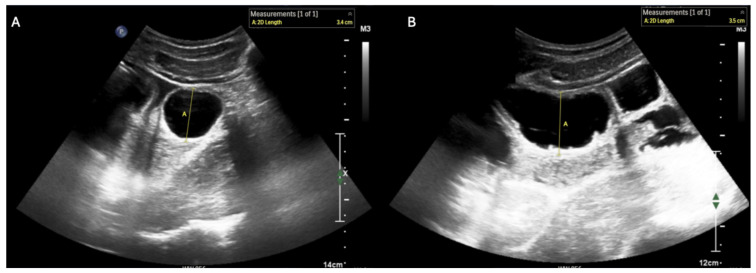
Short- (**A**) and long-axis (**B**) views of small bowel ileus, which has the following characteristics on ultrasound: (i) small bowel outer wall to outer wall diameter ≥ 2.75 cm (with 2.5–2.75 cm representing a borderline value) [8] and (ii) absent peristalsis [10]. Abnormal peristalsis is characterized either by (a) a complete absence of motion of intra-luminal contents or (b) “to-and-fro motion” of the bowel contents with a minimal decrease in size of the small bowel lumen (see Supplementary Video S3 for examples of these dynamic findings) [11].

**Figure 4:**
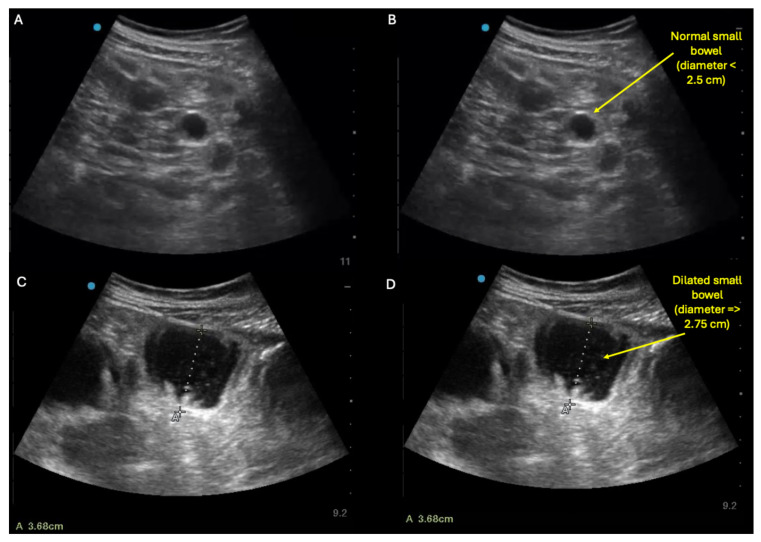
Similarly to small bowel ileus, small bowel obstruction (SBO) on ultrasound will appear as dilated loops of bowel, defined as small bowel diameter ≥ 2.75 cm [11,12,13]. But in contrast to ileus, SBO may also show decompressed small bowel loops in the same abdomen. Thus, the characteristic sonographic finding of small bowel obstruction is concurrent visualization in the same abdomen of both (a) small, decompressed (≤2.5 cm diameter) loops of small bowel (**top panel**: **A**,**B**) and (b) dilated (≥2.75 cm diameter) loops of small bowel lacking peristalsis (**bottom panel**: **C**,**D**) (see also Supplementary Video S4 for examples of these dynamic findings). However, in some cases of SBO, the decompressed loops may not be visualizable with ultrasound, making it difficult to differentiate SBO versus ileus using ultrasound alone. In such cases where ultrasound reveals diffusely dilated loops of small bowel with no decompressed loops and SBO is still suspected, adjunctive imaging such as X-ray and/or CT scan can be pursued.

**Figure 5:**
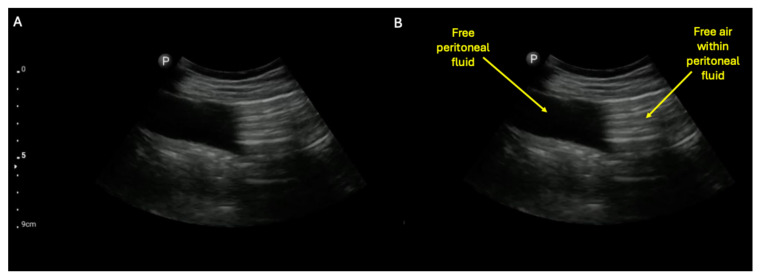
A possible complication of either SBO or ileus is small bowel perforation. The detection of a perforated hollow viscous by ultrasound has been previously described and shown to be diagnostically superior to plain film [14]. A highly suggestive finding is sonographic visualization of pneumoperitoneum within free peritoneal fluid, as shown in the unlabeled (**A**) and labeled (**B**) still images (see also Supplementary Video S4 to visualize dynamic movement of the air within the free peritoneal fluid) [14]. The finding of air within free peritoneal fluid is much more suggestive of pneumoperitoneum than just acoustic shadowing within the abdomen, which can be caused by air-filled loops of bowel alone (e.g., see Figure 6 and Supplementary Video S6).

**Figure 6:**
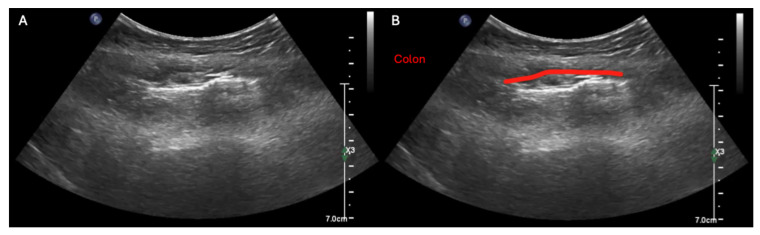
In contrast to obstructive small bowel processes, large bowel dilation can be difficult to definitively rule in with ultrasound alone. This is because the colon (red line) typically appears air-filled whether it is functioning normally or distended, and thus its lumen usually cannot be visualized in its entirety even in cases of severe colonic dilation [15], as shown in this unlabeled (**A**) and labeled (**B**) example of the sonographic findings in severe colonic ileus (see also Supplementary Video S6 for dynamic examples of these sonographic findings). So although a few case reports have described ruling in colonic obstruction with ultrasonography alone [16,17,18], in our experience, large bowel dilation more commonly manifests on ultrasound as diffuse acoustic shadowing within the abdomen with inability to visualize the full lumen of any segment of underlying bowel, which is a rather non-specific finding [5]. Thus, in any patient with abdominal distension, large bowel dilation should be included on the differential if ultrasonography reveals acoustic shadowing throughout the abdomen with inability to see the lumen of the underlying bowel [5]. Further, while POCUS cannot always differentiate small versus large bowel with certainty, several heuristics can be helpful: (i) within the abdomen, the small bowel tends to be centrally located, whereas the large bowel tends to lie peripherally and (ii) the upper limit of normal diameter of the small bowel, colon, and cecum grossly follows the classical 3–6–9 (cm) rule, so bowel that is 3–5 cm in diameter and fluid-filled is much more likely to be dilated small bowel than normal colon because (as mentioned previously) the air found in normal colon usually prevents visualization of the colonic’s entire lumen under either normal or abnormal circumstances [5].

**Figure 7:**
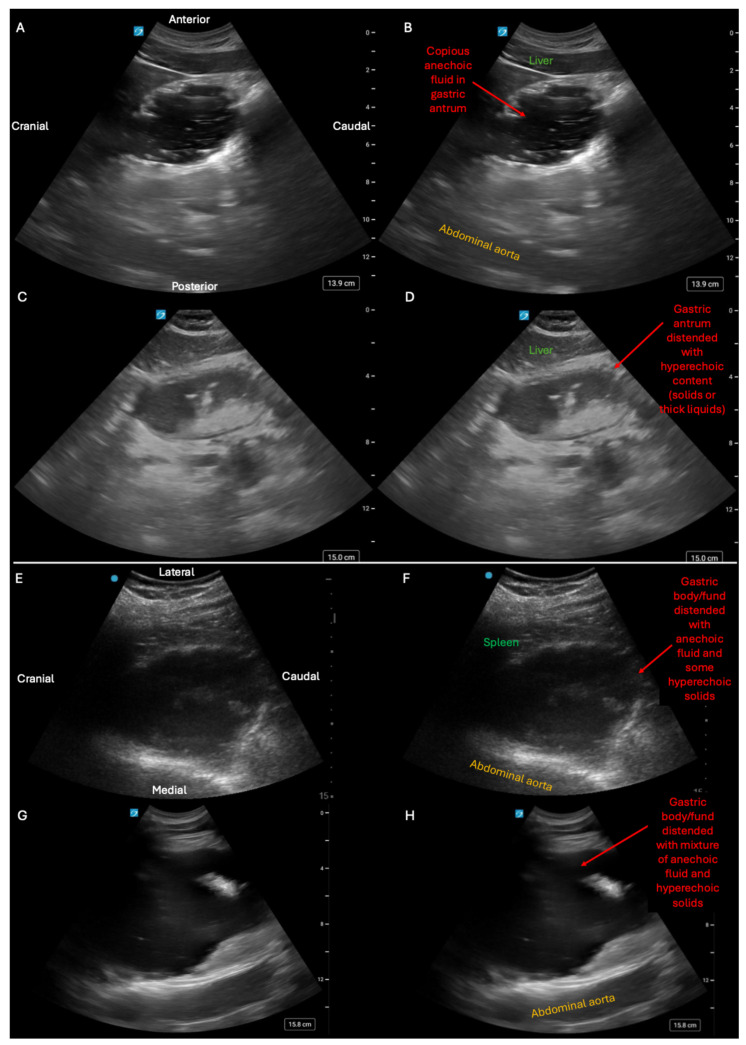
While intestinal ultrasound has been described by several author groups within Emergency, General Surgery, and Internal Medicine [19,20], this work has, in general, remained relatively siloed from parallel work within the field of Anesthesiology to phenotype different states of gastric distension [5,9]. However these two topics can be bridged by simply adding gastric scanning to intestinal evaluation of abdominal distension [5]. While anesthesiologists typically perform gastric ultrasound solely in a single window, we have found a more versatile approach involves gastric evaluation in two locations: subxiphoid evaluation of the gastric antrum (A-D) and left upper quadrant (LUQ) evaluation of the gastric body/fundus (**E**–**H**)) [21]. First row (**A**,**B**) was a subxiphoid view obtained on a patient with normal GI function after intake of clear liquids and illustrates a gastric antrum distended with anechoic fluid. Second row (**C**,**D**) was a subxiphoid view obtained on a patient with gastric outlet obstruction and shows a gastric antrum distended with a mixture of iso-echoic liquids and hyperechoic material, implying some combination of thick liquids and solids. Third row (**E**,**F**) was a LUQ view obtained on a patient with small bowel obstruction causing fluid backup all the way to the stomach, as seen in the gastric body/fundus distended by a mixture anechoic fluid and some hyperechoic solids. Bottom panel (**G**,**H**) was a LUQ view obtained on a patient with normal small bowel function who developed isolated gastroparesis that was visualized as a gastric body/fundus distended with a mixture of anechoic liquids and hyperechoic solids (see also Supplementary Video S7 for corresponding video clips of each stomach). Thus, adding gastric evaluation to intestinal ultrasound allows detection of frank gastric dilation from any process, which can complement intestinal POCUS in at least two situations: (1) for detection of isolated gastroparesis/gastric outlet obstruction as a cause of abdominal distension and (2) for indirect detection of small and/or large bowel dysfunction/obstruction when the intestines are sonographically uninterpretable due to acoustic shadowing from intra-luminal air. Conversely, for anesthesiologists interested in assessing a patient’s aspiration risk prior to surgery, having basic proficiency with intestinal POCUS (Figure 1, Figure 3, Figure 4, Figure 5 and Figure 6) would permit detection of obstructive processes of the small bowel that can materially change anesthetic planning when the stomach itself cannot be visualized. Consequently, greater familiarity by of frontline providers with all the sonographic phenotypes illustrated in this manuscript could help to reduce patient exposure to ionizing radiation and guide management, especially in austere environments where other forms of cross-sectional imaging either require expensive patient transport or are simply not available [22].

**Figure 8:**
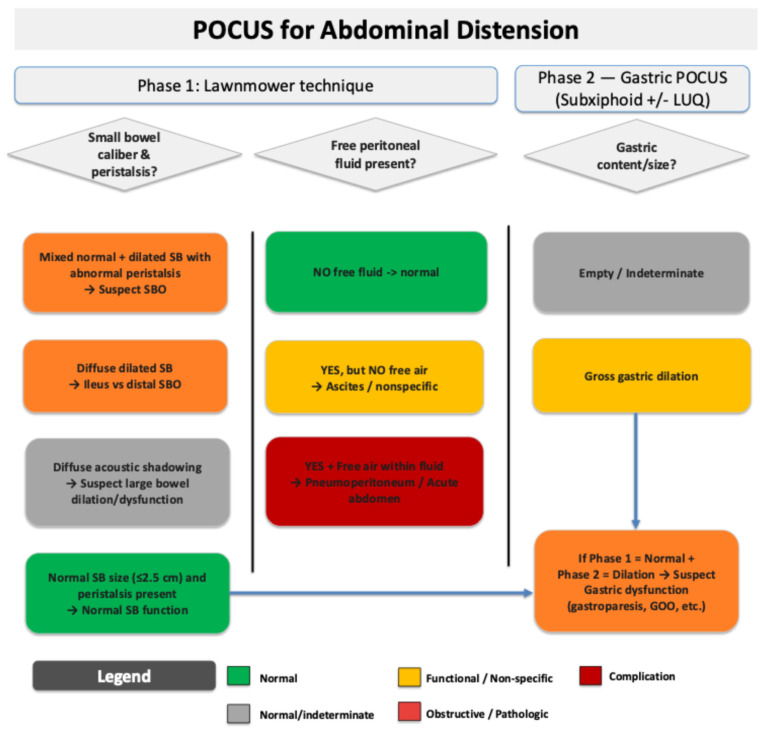
Proposed workflow for integrating intestinal and gastric ultrasound into a two-phase evaluation to screen for the following potential causes of abdominal distension and/or pain: ileus, small bowel obstruction (SBO), and gastric dilation/dysfunction [5]. Notably, while this workflow as a whole has not been studied prospectively, most of the individual components of it have been studied and validated, as shown in Supplementary Table S1. While most of the individual elements of this workflow have been validated in research settings, GI POCUS in general has several important limitations, including but not limited to (i) high operator dependence; (ii) variability in patient body habitus and surgical wounds limiting image quality; (iii) inability to visualize pathology lying deeper to air-filled loops of bowel due to acoustic shadowing; and (iv) overlap in the sonographic appearance of certain bowel conditions such as inflammatory bowel diseases and obstructive pathologies. Some of these limitations might in the future be mitigated by the application of artificial intelligence (AI), which has the potential to shorten the learning curve and increase the diagnostic accuracy of GI POCUS. Toward this goal, several authors have demonstrated the ability of AI to assist with GI POCUS image acquisition and interpretation for certain clinical indications, as shown in Supplementary Table S2.

## Data Availability

The original contributions presented in this study are included in the article. Further inquiries can be directed to the corresponding author.

## Supplementary Materials

The following supporting information can be downloaded at https://www.mdpi.com/article/10.3390/diagnostics15192511/s1: Supplementary Video S1: Video examples of sonographically normal small bowel illustrating the dynamic finding of peristalsis where the small bowel periodically expands and then empties the majority of its contents. Notably, during the expansion phase, the small bowel diameter remains ≤ 2.5 cm under normal circumstances. Supplementary Video S2: Examples of stomach appearance on ultrasound when stomach is grossly empty in subxiphoid and left upper quadrant windows. Supplementary Video S3: Sonographic findings typically seen in ileus: diffusely dilated loops of small bowel either (a) grossly lacking peristalsis or (b) showing to-and-fro peristalsis. Supplementary Video S4: Sonographic findings highly suggestive of small bowel obstruction: concurrent visualization of both (a) dilated and (b) decompressed loops of small bowel within the same abdomen. Supplementary Video S5: Sonographic findings highly suggestive of pneumoperitoneum: free peritoneal fluid containing within it free air moving dynamically. Supplementary Video S6: Video clips of typical finding seen in colonic ileus: diffuse acoustic shadowing throughout the abdomen with inability to see the full lumen of bowel in all or nearly all views. Supplementary Video S7: Video clips of full stomachs seen from the subxiphoid and left upper quadrant windows. Supplementary Table S1: Diagnostic accuracy of POCUS for detection of several gastro-intestinal causes of abdominal distension/pan [23,24,25,26,27,28,29,30]. Supplementary Table S2: Currently published artificial intelligence tools for augmenting GI POCUS [31,32,33,34,35].
