# Supplementary material for: Echoes from Within: Mapping Gastrointestinal Obstruction with Ultrasound
Source: Diagnostics (Basel). 2025 Oct 2;15(19):2511. doi: 10.3390/diagnostics15192511 (PMC12523475; doi:10.3390/diagnostics15192511)
Supplement: Supplementary file 1 [file diagnostics-15-02511-s001.zip › Supplementary Table 1 - diagnostic accuracy of GI POCUS.pdf]

Supplementary Table 1: Diagnostic accuracy of POCUS for detection of several gastro-intestinal causes of abdominal distension / pain

Category	Sensitivity (%)	Specificity (%)	AUC	Notes
**Ileus (post-op)**				Accuracy not well established; diagnosed currently based on qualitative ultrasound features (primarily: dilated bowel and absent peristalsis).[1]
**Small bowel obstruction**	93.0	80.0	0.96	Systematic review/meta-analysis of 21 studies (n=1,977)[2]
**Perforated viscus (pneumoperitoneum)**	91.0	96.0	0.92	Meta-analysis of ultrasound for pneumoperitoneum of 5 studies (N=1,325)[3]
**Gastroparesis**				Not well established; ultrasound can detect delayed emptying but no validated accuracy metrics[4].
Differentiating fasted vs non-fasted stomach				*Non-fasted defined in each study as noted below:*
**Healthy volunteers**	100.0	98.0		Solids or estimated GRV > 1.5 mL/kg[5]
**Pregnancy (3rd trimester)**	80.0	67.0	0.82	Gastric antral CSA ≥9.6 cm^2[6]
**Adults >60**	75.0	100.0		Gastric antral CSA ≥10.4 cm^2[7]
**All patients**	95.0	88.0	0.97	Heterogeneous definitions[8]

- **AUC** – Area Under the Receiver Operator Curve
- **CSA** – Cross-Sectional Area

References:

1. Hollerweger, A.; Maconi, G.; Ripolles, T.; Nylund, K.; Higginson, A.; Serra, C.; Dietrich, C.F.; Dirks, K.; Gilja, O.H. Gastrointestinal Ultrasound (GIUS) in Intestinal Emergencies - An EFSUMB Position Paper. *Ultraschall Med* **2020**, *41*, 646–657, doi:10.1055/a-1147-1295.
2. Motavaselian, M.; Farrokhi, M.; Jafari Khouzani, P.; Moghadam Fard, A.; Daeizadeh, F.; Pourrahimi, M.; Mehrabani, R.; Amani-Beni, R.; Farrokhi, M.; Jalayer Sarnaghy, F.; et al. Diagnostic Performance of Ultrasonography for Identification of Small Bowel Obstruction; a Systematic Review and Meta-analysis. *Arch Acad Emerg Med* **2024**, *12*, e33, doi:10.22037/aaem.v12i1.2265.
3. Jiang, L.; Wu, J.; Feng, X. The value of ultrasound in diagnosis of pneumoperitoneum in emergent or critical conditions: A meta-analysis. *Hong Kong Journal of Emergency Medicine* **2019**, *26*, 111–117, doi:10.1177/1024907918805668.

4. Steinsvik, E.K.; Sangnes, D.A.; Søfteland, E.; Biermann, M.; Assmus, J.; Dimcevski, G.; Gilja, O.H.; Hausken, T. Gastric function in diabetic gastroparesis assessed by ultrasound and scintigraphy. *Neurogastroenterol Motil* **2022**, *34*, e14235, doi:10.1111/nmo.14235.
5. Kruisselbrink, R.; Gharapetian, A.; Chaparro, L.E.; Ami, N.; Richler, D.; Chan, V.W.S.; Perlas, A. Diagnostic Accuracy of Point-of-Care Gastric Ultrasound. *Anesthesia and analgesia* **2019**, *128*, 89–95, doi:10.1213/ane.0000000000003372.
6. Arzola, C.; Perlas, A.; Siddiqui, N.T.; Downey, K.; Ye, X.Y.; Carvalho, J.C.A. Gastric ultrasound in the third trimester of pregnancy: a randomised controlled trial to develop a predictive model of volume assessment. *Anaesthesia* **2018**, *73*, 295–303, doi:10.1111/anae.14131.
7. Wang, J.; Shuai, Y.; Cheng, Y.; Zhang, Y. Ultrasound assessment of gastric residual volume in patients over 60 years of age undergoing gastroscopy under sedation: a prospective cohort study. *Canadian journal of anaesthesia = Journal canadien d'anesthesie* **2023**, *70*, 1315–1322, doi:10.1007/s12630-023-02523-1.
8. Pan, X.; Chai, J.; Gao, X.; Li, S.; Liu, J.; Li, L.; Li, Y.; Li, Z. Diagnostic performance of ultrasound in the assessment of gastric contents: a meta-analysis and systematic review. *Insights Imaging* **2024**, *15*, 98, doi:10.1186/s13244-024-01665-0.
