# Supplementary material for: Echoes from Within: Mapping Gastrointestinal Obstruction with Ultrasound
Source: Diagnostics (Basel). 2025 Oct 2;15(19):2511. doi: 10.3390/diagnostics15192511 (PMC12523475; doi:10.3390/diagnostics15192511)
Supplement: Supplementary file 1 [file diagnostics-15-02511-s001.zip › Supplementary Table 2 - Currently published artificial intelligence tools for augmenting GI POCUS.pdf]

Supplementary Table 2: Currently published artificial intelligence tools for augmenting GI POCUS

Domain	AI Application	Population / Dataset	Performance	Notes
**Gastric POCUS**	Deep learning segmentation & motility analysis of gastric antrum (CSA + rhythm)	Internal 3,119 images, external 1,346 images	mDice 87.4% (IOU 77.6%); external mDice 86.8% (IOU 76.3%); ICC vs humans 0.81	Automates antrum localization, CSA measurement, and peristaltic rhythm tracking[1]
**Pediatric intestinal ultrasound (intussusception)**	CIDNet deep learning classifier for ileocolic intussusception on ultrasound images	9,999 US images from 4,154 children (training/validation)	AUC 0.973; comparable to senior radiologists in reader study	Outperformed other deep learning models; may support non-expert clinicians[2]
**Bedside device auto-tools**	Vendor-provided AI tools (e.g., Auto Gastric Antrum, Smart-IVC, Auto-B-line)	Integrated into handheld devices	Not benchmarked in large trials; designed for real-time acquisition guidance	Intended to reduce operator dependence and speed acquisition/measurement[3]
**Intestinal ultrasound (Pediatric IBD)**	AI-assisted BWT segmentation and classification abnormal vs normal	260 children; 4,565 IUS images; annotated subset 612 images	Sensitivity 90.3%, Specificity 93.7%; ICC 0.942 vs manual	BWT cutoff 2 mm; strong agreement with manual; pediatric IBD focus[4]
**Intestinal ultrasound (Crohn's disease)**	CNN classifier detecting bowel wall thickening (>3 mm) vs normal	1,008 images (805 train / 203 validation)	Sensitivity 86.4%, Specificity 94.0%, AUC 0.978	Crohn's disease; single-center dataset[5]

- **AUC** – Area Under the Receiver Operator Curve
- **BWT** – Bowel Wall Thickness
- **CNN** – Convolutional Neural Network
- **CSA** – Cross-Sectional Area
- **IBD** – Inflammatory Bowel Disease
- **ICC** – Intraclass Correlation Coefficient
- **IOU** – Intersection over Union
- **IUS** – Intestinal Ultrasound
- **mDice** – Mean Dice similarity coefficient

References:

1. Zou, T.; He, H.; Yang, J.; Wu, Y.; Lv, C.; Zhao, L.; Yin, W. Artificial intelligence real-time automated recognition of the gastric antrum cross-sectional area and motility rhythm via bedside ultrasound: a pilot study. *Sci Rep* **2025**, *15*, 13883, doi:10.1038/s41598-025-98974-1.
2. Chen, X.; You, G.; Chen, Q.; Zhang, X.; Wang, N.; He, X.; Zhu, L.; Li, Z.; Liu, C.; Yao, S.; et al. Development and evaluation of an artificial intelligence system for children intussusception diagnosis using ultrasound images. *iScience* **2023**, *26*, 106456, doi:10.1016/j.isci.2023.106456.
3. Cui, X.W.; Goudie, A.; Blaivas, M.; Chai, Y.J.; Chammas, M.C.; Dong, Y.; Stewart, J.; Jiang, T.A.; Liang, P.; Sehgal, C.M.; et al. WFUMB Commentary Paper on Artificial intelligence in Medical Ultrasound Imaging. *Ultrasound Med Biol* **2025**, *51*, 428–438, doi:10.1016/j.ultrasmedbio.2024.10.016.
4. Kumaralingam, L.; Le May, K.; Dang, V.B.; Alavi, J.; Huynh, H.Q.; Le, L.H. Artificial intelligence-assisted approach to assessing bowel wall thickness in pediatric inflammatory bowel disease using intestinal ultrasound images. *J Crohns Colitis* **2025**, *19*, doi:10.1093/ecco-jcc/jjaf037.
5. Carter, D.; Albshesh, A.; Shimon, C.; Segal, B.; Yershov, A.; Kopylov, U.; Meyers, A.; Brzezinski, R.Y.; Ben Horin, S.; Hoffer, O. Automatized Detection of Crohn's Disease in Intestinal Ultrasound Using Convolutional Neural Network. *Inflamm Bowel Dis* **2023**, *29*, 1901–1906, doi:10.1093/ibd/izad014.
